# Impact of increased *Porphyromonas gingivalis* peptidylarginine deiminase (PPAD) T2 variant allele on oral microbiota composition and severity of chronic periodontitis

**DOI:** 10.1080/20002297.2025.2479903

**Published:** 2025-03-20

**Authors:** Marta Kaminska, Noemie A.M. Dudzinska, Tülay Yucel-Lindberg, Birgitta Söder, Aswathy Narayanan, Jan Potempa, Piotr M. Mydel

**Affiliations:** a Broegelmann Research Laboratory, Department of Clinical Science, Faculty of Medicine, University of Bergen, Bergen, Norway; b Division of Pediatric Dentistry, Department of Dental Medicine, Karolinska Institutet, Stockholm, Sweden; c Department of Microbiology, Faculty of Biochemistry, Biophysics and Biotechnology, Jagiellonian University, Krakow, Poland; d Division of Periodontology, Department of Dental Medicine, Karolinska Institutet, Stockholm, Sweden; e Division of Infectious Diseases, Department of Medicine Huddinge Karolinska Institutet, Stockholm, Sweden; f Department of Oral Immunology and Infectious Diseases, University of Louisville School of Dentistry, Louisville, KY, USA

**Keywords:** Periodontitis, microbiota, PPAD, citrullination, PPAD variant, PPAD super-active variant, PPAD-T2

## Abstract

**Background:**

*Porphyromonas gingivalis* (Pg) is a keystone pathogen in periodontitis, encoding a unique peptidyl arginine deiminase (PPAD) linked to protein citrullination, a process associated with rheumatoid arthritis (RA). Recently, we identified a super-active PPAD variant (T2) in *Pg* isolates. Here, we evaluated if the presence of the super-active T2 variant of PPAD affects the salivary microbiome, the severity of chronic periodontitis (CP), and subsequently CP’s causative association with RA onset/progression.

**Patients/Materials and Methods:**

We examined 56 CP patients and 36 healthy volunteers. *Pg* and *Tannerella forsythia* counts were measured *via* RT-PCR, and PPAD variant was typed *via* PCR. 16S rRNA from salivary DNA sequencing characterized microbiota composition, while CP severity was assessed through bleeding on probing (BoP), clinical attachment loss (CAL), and pocket depth (PD) parameters.

**Results:**

CP patients exhibited higher *Pg* and *T.* *forsythia* counts, with 30.7% harbouring the PPAD-T2 variant, compared to only one healthy volunteer. Clinical CP parameters were unaffected by the PPAD variant. However, PPAD-T2 influenced oral microbiota composition, enriching certain genera.

**Conclusion:**

While the PPAD variant did not affect CP severity, it influenced oral microbiota composition. Further research is needed to understand citrullination’s role in oral microbiota and chronic inflammatory disease development.

## Introduction

The influence of the human microbiota on health and disease has garnered considerable and increasing interest in recent years. An emerging paradigm suggests that a dysbiotic shift in the composition of microbial flora in various compartments of the human body could result in serious pathological consequences, ranging from metabolic diseases and obesity to cancer and mental disorders [1–4].

While the gut microbiota has been the primary focus due to its high microbial density, the effect on human health and disease of the second largest and most diverse microbial community in the human body, the oral microbiota, has only recently begun to be explored.

Accumulation of dysbiotic oral microbiota is the keystone of chronic periodontitis (CP) [5]. CP has been associated with a spectrum of debilitating disorders, including rheumatoid arthritis (RA), Alzheimer’s disease (AD) and cardiovascular diseases [6]. A causative link between RA and CP has been proposed to involve the host immune response to specific periodontal pathogens in susceptible individuals [7]. The current literature implicates the breakdown of immunotolerance to citrullinated epitopes occurring in inflamed mucosal surfaces resulting in production of anti-citrullinated protein antibodies (ACPA) as a pivotal event in RA initiation [8]. ACPA, presently used as biomarkers for RA, recognize an array of human citrullinated proteins (including filaggrin, fibrinogen, alpha-enolase, histones, and vimentin) [9], generated through post-translational modification of arginine to citrulline catalysed by the family of peptidylarginine deiminases (PADs). However, the mechanisms triggering ACPA production are still unclear.

It is generally accepted that a relatively small consortium of bacteria (*Porphyromonas gingivalis*, *Treponema denticola,* and *Tannerella forsythia*), described as the ‘red complex’, is strongly associated with the pathological changes in the periodontium [10]. From the host’s perspective these anaerobic, Gram-negative bacteria are intrusive in the very diverse community of microbial biofilm on the subgingival tooth surface, and their proliferation significantly changes the composition of the community (microbiota shift). The proliferation of the ‘red complex’ species initiates a chronic inflammation of the periodontium, which, if left untreated, over the years destroys the tooth-supporting structures as a consequence of the futile attempt of the host’s innate immune response to eradicate microbial invaders that thrive in the inflammatory milieu [6,11,12].

Since PPAD was first isolated and characterized in 1999 [13], the enzyme has been suspected to contribute to *P. gingivalis* virulence. However, only recently have the mechanisms of PPAD’s involvement in the pathogenicity of PD been elucidated. PPAD-driven citrullination of C-terminal residues in epidermal growth factor [14] and C5a anaphylatoxin [15] can contribute to periodontal tissue damage and attenuation of innate immune responses, respectively. Additionally, the citrullinome of *P. gingivalis* influences neutrophil function [16], limits the formation of *P. gingivalis* biofilm [17], and alters the transcriptome of epithelial cells [18]. Citrullinated proteins of *P. gingivalis* stimulate human gingival fibroblasts to produce prostaglandin E2 (PGE2), a potent inflammatory mediator of bone resorption implicated in the pathogenesis of CP and RA [19]. The concerted action of *P. gingivalis* virulence factors – arginine-specific gingipains generating C-terminal arginine residues, which are subsequently modified by PPAD – is considered pivotal in breaking the immunotolerance. This leads to the production of specific anti-citrullinated protein antibodies (ACPA) directly implicated in the development of RA [20]. The importance of *P. gingivalis*-induced citrullination in oral mucosa for RA development was further confirmed using transcriptomic data of blood samples from RA patients with and without periodontal disease. CP-related repeated breaches of the oral mucosa and subsequent release of citrullinated host and bacterial proteins into circulation activate the subsets of inflammatory monocytes observed in inflamed synovium and blood of RA patients. Moreover, this event also activates ACPA-positive B cells, promoting affinity maturation and epitope spreading to citrullinated human antigens [21].

Two allelic forms of the *ppad* gene among *P. gingivalis* clinical isolates were recently described [22], one variant encoding a super-active isoform of the enzyme binding substrates with higher affinity (PPAD‐T2) and two-fold higher cell‐associated citrullinating activity. This discovery has prompted us to evaluate whether the severity of CP and its subsequent link to RA progression is associated with the occurrence of the super-active T2 variant of PPAD. Since the growth of *P. gingivalis* is inhibited at low pH [23,24], increased citrullination in combination with amino acid fermentation could create a highly favourable environment for its survival, thus affecting the composition of the oral microbiota. Additionally, the T2 variant could significantly impact the level of citrullination in the subgingival pocket. While numerous studies focus on the association of *P. gingivalis* with RA, very little is known about the influence of citrullinated milieu on the oral microbiome composition and the effect of citrullination on the progression of CP. Therefore, using salivary DNA profiles from patients with CP and healthy controls, we aim to characterize and quantify the impact of the presence of PPAD-T2 on oral microbiome composition, the clinical picture of CP, and its possible association with RA.

## Materials and methods

### Ethical considerations

The study, which involved the collection of saliva samples, received approval from the Regional Ethical Review Board in Stockholm (2009/792–31/4, 2014/1588–32/3), and all participants provided written informed consent.

### Dental clinical examination

In the present study, a total of 92 individuals were included, divided into two groups, a periodontitis group (*n* = 56) and a non-periodontitis (*n* = 36). The clinical full-mouth examination included evaluation of plaque index (PLI), bleeding on probing (BOP), clinical attachment loss (CAL) and pocket depth (PD). Participants exhibiting at least one site with a PD of 5 mm or greater, CAL of 3 mm or more, and radiographic evidence of bone loss were diagnosed with periodontitis, as previously described [25].

### Saliva sample collection

In connection with a clinical dental examination, saliva samples were collected for 16S rRNA sequencing and Real-time (rt) PCR analysis. Whereas there is no consensus on whether saliva sampling is representative of the microbial composition present in subgingival or supragingival dental plaque [26,27], it is commonly used due to its accessibility. Here, we used stimulated saliva samples thought to better represent the oral microbiota than the unstimulated saliva [26,27]. Stimulated saliva samples were obtained by having each participant chew on paraffin wax (1 gram, Ivoclar Vivadent, Liechtenstein) for 5 minutes. The collected samples were stored in sterile 50 mL Falcon tubes and immediately frozen at −20°C until processing, including centrifugation at 500 × g for 10 minutes at 5°C. The resulting supernatants were collected, stored in Eppendorf tubes at −80°C, and reserved for further processing and analysis as described previously [28].

### DNA isolation

Stimulated saliva was vortexed briefly. Four hundred µl was transferred to a fresh tube and centrifuged for 15 min at 10,000 rpm, 4°C. Then, the supernatant was discarded, and the pellet was suspended in 200 µl PBS. DNA was isolated using a commercially available kit QIAamp DNA mini kit (Qiagen) according to the producer’s instructions. After elution, the concentration of isolated DNA was measured using NanoDrop, and DNA was frozen until further processing.

### 16S rRNA gene library preparation and sequencing

V3-V4 variable regions of the 16S rRNA gene were amplified using 2 ng extracted DNA from each saliva sample. The PCR reactions were prepared with final concentrations of 1× KAPA HotStart ReadyMix (KAPA Biosystems, Wilmington, MA, USA), 1.0 µM forward primer (5’- CCTAHGGGRBGCAGCAG-3’), 1.0 µM reverse primer (5’-GACTACHVGGGTATCTAATCC-3’), and 0.1 ng/µl DNA. The PCR amplification was carried out with the following temperature profile: an initial denaturation step at 98°C for 2 minutes, followed by 26 cycles of denaturation at 98°C for 20 seconds, annealing at 54°C for 20 seconds, and extension at 72°C for 15 seconds. The final elongation step was performed at 72°C for 2 minutes. The libraries were indexed using 12 µl of the purified sample, 0.4 µM forward and 0.4 µM reverse indexing primer and KAPA HotStart ReadyMix. After the PCR reaction, equimolar amounts of indexed samples were combined to create two separate pools. These pools of samples were then sequenced in separate runs on the Illumina MiSeq platform (Illumina Inc, San Diego, CA, USA) at NGI/SciLifeLab (Stockholm, Sweden) as previously described [28].

### Sequence data processing and analysis of data

To extract biological sequences from the reads, the DADA2 pipeline was employed. This tool enables inference of biological sequences from amplicon reads by modeling Illumina sequencing errors. After initial trimming and filtering to remove low-quality reads and sequencing artifacts (such as primers, PhiX, and chimeras), the retained reads were denoised using DADA2. Next, paired-end reads were merged with a 30-bp overlap with no mismatches, and low-count taxa were filtered out by including only sequence types (STs) with at least one read present in at least 5% of the samples. Finally, a phylogenetic tree was constructed using Quantitative Insights into Microbial Ecology version 2 (QIIME2). Amplicon sequence variants (ASVs) were generated using QIIME2 for functional interpretation of the microbiota [29]. All statistical analyses were conducted using the R package (version 3.2.1) with the phyloseq library (McMurdie and Holmes, 2013). The DADA2 table, sample data, and taxonomic table were combined into a phyloseq object to study the beta-diversity of the samples. Beta-diversity was estimated using the ordinate R function and visualized using the plot ordination R function. The clustering of the samples was visualized using a principal coordinate analysis (PCoA) plot based on Bray–Curtis distance.

### Real-time PCR

Equal volumes of salivary DNA template (1 µl) were pipetted onto a 96-well plate. Next, 7.5 µl of PowerUP SYBR Green Master Mix 2× (ThermoFisher), 0.5 µl of forward and reverse primers (final primer concentration during reaction: 0.33 µM) and water was added to the final volume of 15 µl. PCR reaction was performed in the following conditions: for 16S reactions: 2 min at 95°C, 40 cycles of 15s at 95°C and 1 min at 60°C, with final elongation 2 min at 60°C; for PPAD typing reactions: 3 min at 95°C, 40 cycles of 30s at 95°C, 30s at 65°C, and 15s at 72°C, with final elongation 5 min at 72°C. Reaction product quality was analysed *via* melt curve.

Oligonucleotide sequences of primers used in the study were as follows:

*P. gingivalis* 16S Forward: 5’-AGGCAGCTTGCCATACTGCG-3’,

Reverse: 5’-ACTGTTAGCAACTACCGATGT-3’;

*T. forsythia* 16S Forward: 5’-GCGTATGTAACCTGCCCGCA-3’,

Reverse: 5’-TGCTTCAGTGTCAGTTATACCT-3’;

*P. intermedia* 16S Forward: 5’-GACCAAAGATTCATCGGTGGAG-3’,

Reverse: 5’-CACGCTACTTGGCTGGTTC-3’;

*P. gingivalis* PPAD T1 and T2 Forward: 5’-GAACAAAGTAGGTCTCGTGGAC-3’,

T1 Reverse: 5’-TCCACATGGTTGATATATTCGCC-3’,

T2 Reverse: 5’- CAGTCCACATGGTCGATATAAGTATT-3’.

Colony forming units (CFU) of *P. gingivalis* and *T. forsythia* were calculated using threshold values for standard curve obtained using DNA preparations of known amount of bacteria.

### Statistical analysis

Results are presented as mean ± standard error of the mean unless indicated otherwise. One-way ANOVA was employed to check the statistical significance of loads of bacteria between the studied groups. Biofilm composition differences concerning PPAD type, or an association between PPAD type and clinical measurements were assessed with the Mann-Whitney test, Kruskal-Wallis test, or unpaired *t*-test. Statistical analysis was performed using GraphPad Prism version 10.1.2 (Boston, Massachusetts USA).

## Results

### PPAD-T2 has a prevalence of around 30% among *P. gingivalis*-positive patients

Using DNA extracted from saliva samples from CP patients, we evaluated the *P. gingivalis* load and predominance of the PPAD-T2 gene with real-time PCR. Overall, we have screened the samples from 56 CP patients and 36 healthy volunteers. The demographics of the participants have been described in Table 1.

**Table 1.** Demographics of the patients and healthy volunteers included in the study.

Variable		Healthy * n* = 36		Periodontitis * n* = 56		p value
		*n (percent)*	*Mean ± SD*	*n (percent)*	*Mean ± SD*	
Age			49.8 ± 2.8		48.4 ± 3.6	0.0528^I^
Gender	Male	18 (50%)		31 (55.4%)		0.6717^II^
	Female	18 (50%)		25 (44.6%)		
Smokers		7 (19.4%)		29 (51.8%)		0.0022^II^
Co-morbidities	Heart disease	10 (27.8%)		18 (32.1%)		0.8169^II^
	CVD (other)	0		3 (5.4%)		0.2776^II^
Clinical measurements	PLI		0.50 ± 0.27		0.86 ± 0.43	<0.001^III^
	BoP [%]		38 ± 17		59 ± 22	<0.0001^III^
	CAL [mm]		2.8 ± 0.41		3.5 ± 0.76	<0.0001^III^
	PD = 4 mm [nr of sites]		9 ± 7		21 ± 10	<0.0001^III^
	PD > 5 mm [nr of sites]		0		10 ± 13	<0.0001^III^

SD ─ standard deviation, CVD – cardiovascular diseases other than heart diseases, BoP – bleeding on probing, CAL – clinical attachment loss, PD – pocket depth, PLI- Plaque index, *p* values have been evaluated with student t-test (^I^), Fisher’s exact test (^II^) or Mann-Whitney test (^III^).

Among healthy controls, *P. gingivalis* was detected in 1 case (2.8%) while CP patients were positive in 25 cases (44.6%). All *P. gingivalis*-positive samples from CP patients and controls were then typed for PPAD variants (T1 and T2) *via* RT-PCR. Overall, we have found that the PPAD-T2 variant was present in 32% of cases while PPAD-T1 in 68% (Table 2). We have not detected any case of both PPAD variants’ simultaneous presence in any of the subjects.

**Table 2.** *P. gingivalis* positivity and PPAD variant distribution in the cohort and in the context of sex.

Variable		Healthy *n* = 36	Periodontitis *n* = 56		Male * n* = 31	Female *n* = 25	
		*n (percent)*	*n (percent)*	p value	*n (percent)*	*n (percent)*	p value
*P. gingivalis*-positivity		1 (2.8%)	25 (44.6%)	<0.0001	11 (35.5%)	15 (60.0%)	0.2468
PPAD (of *P. gingivalis*-positive)	Variant T1	1 (100%)	17 (68%)	>0.9999	9 (81.8%)	9 (60%)	0.3945
	Variant T2	0	8 (32%)		2 (18.2%)	6 (40%)	

*p* values have been evaluated with Fisher’s exact test.

*P. gingivalis* positivity or PPAD variant type were not influenced by smoking (among T2-positive patients 50% were smokers; data not shown). However, the majority of the PPAD-T2 variant strains were carried by female CP patients (among T2-positive patients 75% were females; Table 2).

**Figure 1.**
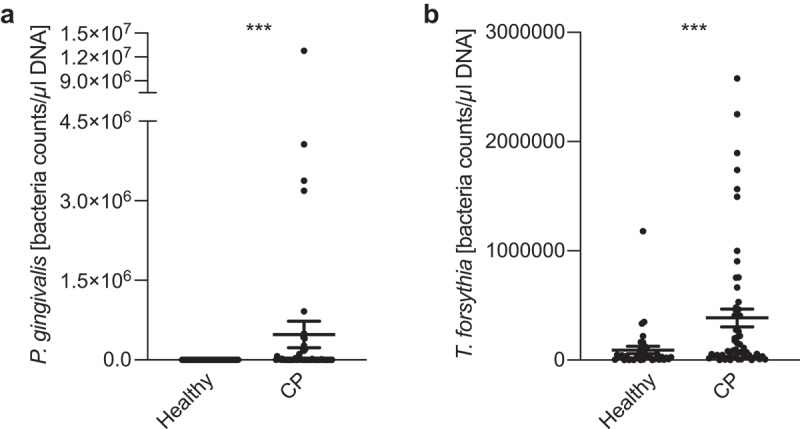
*P. gingivalis* (a) and *T. forsythia* (b) counts in healthy volunteers and patients with CP.

Not surprisingly, in almost all the CP patients positive for *P. gingivalis* its loads were several orders of magnitude higher than in a positive healthy volunteer. As *T. forsythia* is frequently isolated together with *P. gingivalis*, especially from patients with the active state of periodontitis [30], we also evaluated its presence in saliva and relative abundance. While 34 out of 36 tested controls were positive for *T. forsythia*, they had relatively low load of the bacteria (97748 ± 36054) as compared to the CP (55 of 56) group (392333 ± 81663) (Figure 1a,b).

DNA was isolated from stimulated saliva. Equal volumes were used in rtPCR to assess the numbers of *P. gingivalis* or *T. forsythia*. CFU – colony-forming unit. Statistical significance between group was evaluated *via* Mann-Whitney test. *p < .05, **p < .01, ***p < .001.

**Figure 2.**
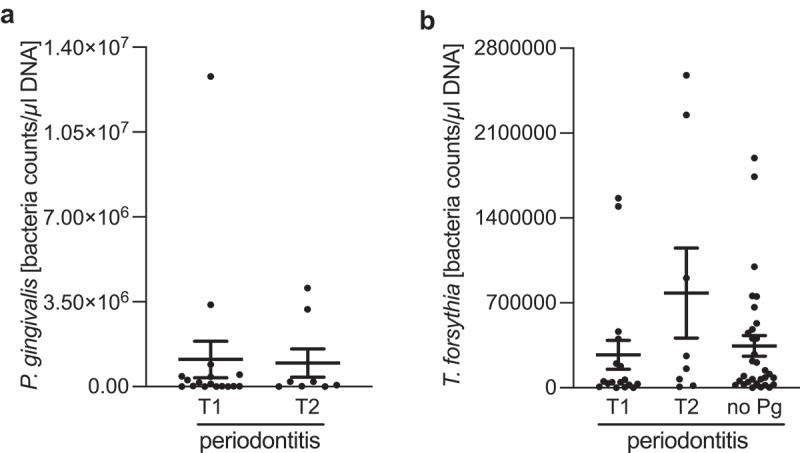
Relationship between the bacterial loads of *P. gingivalis* (a) and *T. forsythia* (b) and PPAD variants.

No significant associations were found between the bacterial load of neither *P. gingivalis* nor *T. forsythia* depending on the PPAD type among CP patients (Figure 2b).

DNA was isolated from stimulated saliva. Equal volumes were used in rtPCR to type PPAD variant using primer pairs designed to allow selective amplification of the chosen variant. T1 ─ PPAD-T1 variant, T2 ─ PPAD-T2 variant. Statistical significance between group was evaluated *via* Kruskal-Wallis test, with Dunn’s post-hoc analysis. *p < .05.

### The super-active form of PPAD had no impact on the severity of CP

**Figure 3.**
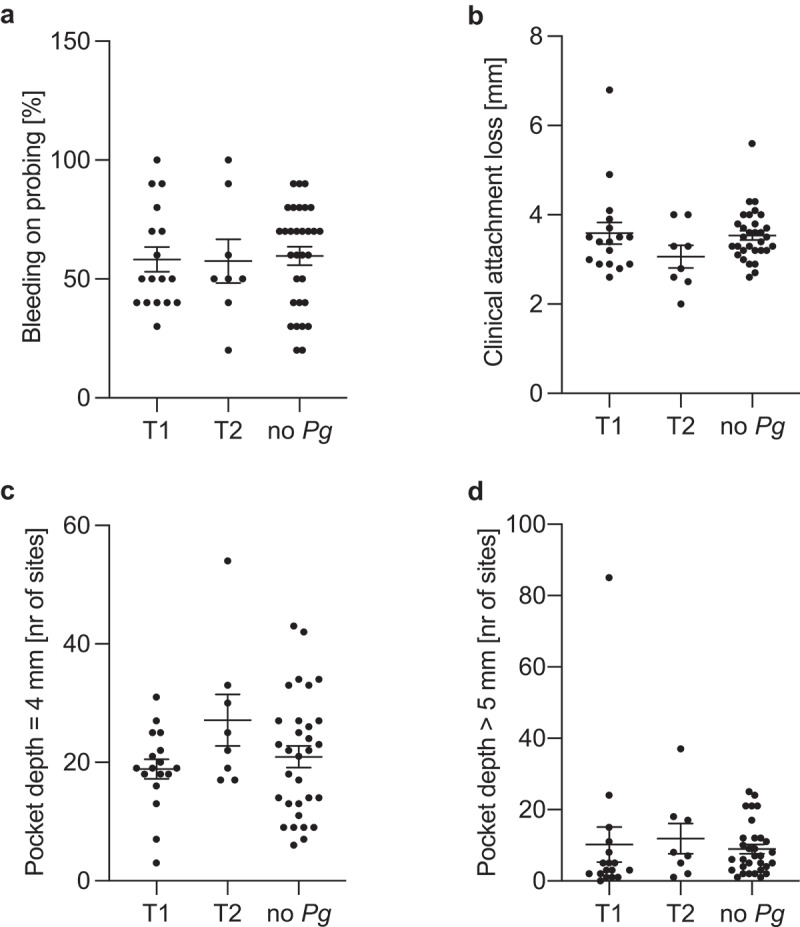
Clinical manifestation of CP depending on the variant of PPAD. (a) bleeding on probing [%], (b) clinical attachment loss [mm], (c) PD = 4 mm [number of sites], and (d) PD >5 mm [number of sites]. Statistical significance between group was evaluated *via* Kruskal-Wallis test, with Dunn’s post-hoc analysis. *p < .05.

To determine if the type of PPAD expressed by *P. gingivalis* influences its virulence and has an impact on CP progression and severity, we have evaluated clinical parameters in patients based on the type of strain present. None of the evaluated clinical parameters of CP (BoP, CAL) at selected sites were influenced by the variant of PPAD. Moreover, the number of sites with PD = 4 mm and ≥5 mm remained the same in both groups (Figure 3c,d).

### The salivary microbiome is affected by the PPAD variant

**Figure 4.**
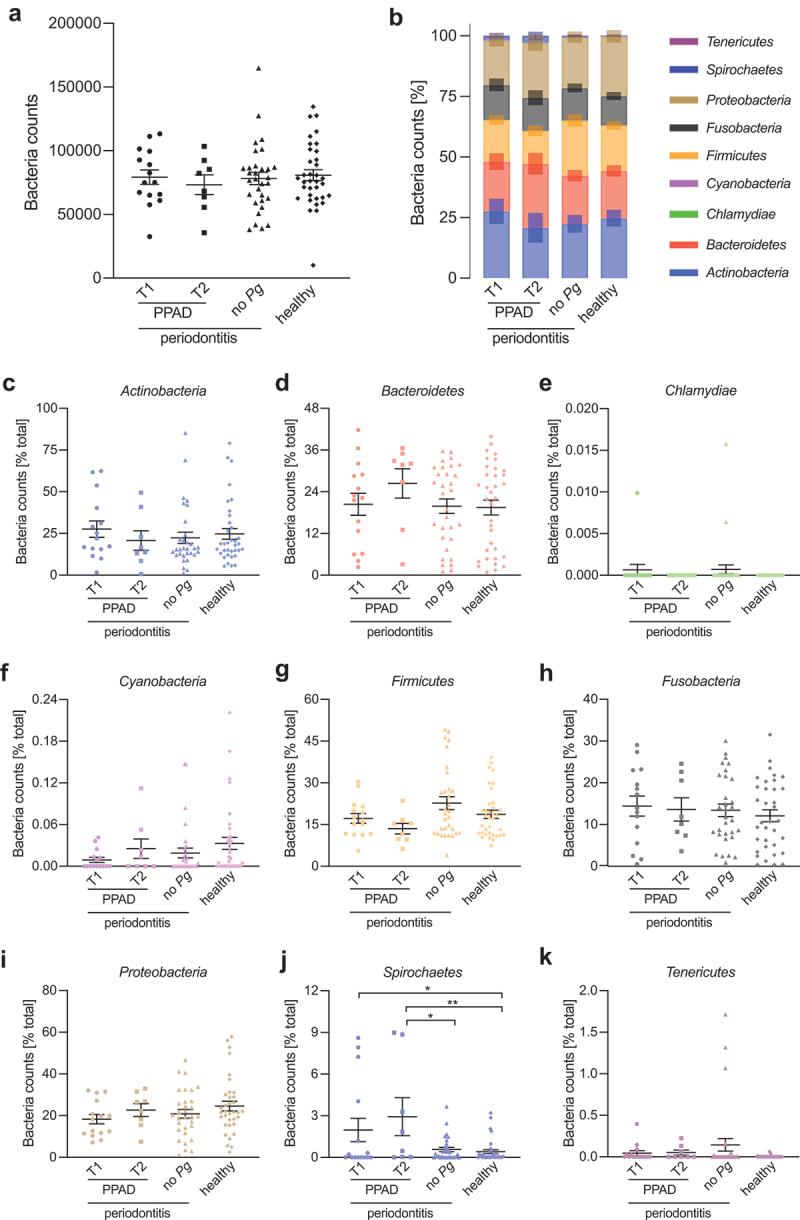
Influence of PPAD variants on salivary microbiota: (a) bacteria counts, (b) biofilm composition as % of total bacteria counts, and abundance of (c) *Actinobacteria*, (d) *Bacteroidetes*, (e) *Chlamydiae*, (f) *Cyanobacteria*, (g) *Firmicutes*, (h) *Fusobacteria*, (i) *Proteobacteria*, (j) *Spirochaetes*, (k) *Tenericutes*. Salivary microbiota composition was characterized *via* 16S rRNA gene sequencing. Statistical significance between groups was evaluated with One-way ANOVA, with Tukey’s post-hoc analysis. *p < .05, **p < .01, ***p < .001.

Although only a few genera have been identified as particularly important in CP pathogenesis, the entire dysbiotic oral microbiota is a driving force behind the progression of periodontitis. The increased citrullination *via* the PPAD-T2 variant in combination with amino acid fermentation could generate a highly favorable environment for *P. gingivalis* survival, affect the oral microbiota structure, and shift the salivary microbiome into more pathogenic. That phenomenon could subsequently affect the odds of the chronic inflammatory diseases’ development at a later stage in susceptible patients. Using the 16S rRNA gene amplicon sequencing data we evaluated the salivary microbiome composition depending on the PPAD variant found in the CP patients’ microbiota. The study cohort comprised 92 participants separated into two groups: the periodontitis group (*n* = 56) and the non-periodontitis group (*n* = 36). Differences in beta-diversity were visualized using PCoA plot based on Bray-Curtis distances between the samples of healthy and periodontitis groups (Supplementary Figure S1A). During the analysis we further divided CP patients into 3 groups ─ *P. gingivalis*-negative, *P. gingivalis* T1, and *P. gingivalis* T2 to fully evaluate the impact of expression of PPAD variants on microbiota composition. Differences between PPAD-T1 and PPAD-T2 subpopulations among periodontitis group were represented using PCoA plot based on Bray-Curtis distances (Supplementary Figure S1B). We analysed the subgingival microbial composition at different taxonomic levels, including phylum, family, and genus. In total, 12 operational taxonomic units (OTU) at the phylum level were identified. The distribution pattern of the top 9 phyla (comprising over 99.5% of the total counts) in each group, including PPAD-T1 and PPAD-T2 group, is shown in Figure 4b,c,k.

The most predominant phyla across all samples were *Firmicutes* (median relative abundance 38.2%), *Bacteroidetes* (21.1%), and *Proteobacteria* (17.9%). The relative abundance among all the groups was similar independently of the presence or absence of the disease and *P. gingivalis* (Figure 4b). Notably, the number of spirochetes, although a relatively small group (less than 3% of total bacteria), was significantly increased in *P. gingivalis*-positive individuals, independently from their clinical status. In contrast to the previous reports, we have not seen statistically important increase in the abundance of *Bacteroidetes* and *Firmicutes* in periodontitis [31,32]. At the genus level, periodontitis patients positive for *P. gingivalis* had a higher abundance of *Candidatus Saccharibacteria, Schwartzia*, and *Peptostreptococcus* while significantly lower numbers of *Streptococcus*.

Although there was no association between the PPAD type present and the clinical picture of the CP and the PPAD variant exerted no effect on the relative abundance distribution across the analysed phyla, class, order or family (Figure 4b), we have found several differences in the genera and families which suggests the contribution of PPAD activity on the microbiota composition.

**Figure 5.**
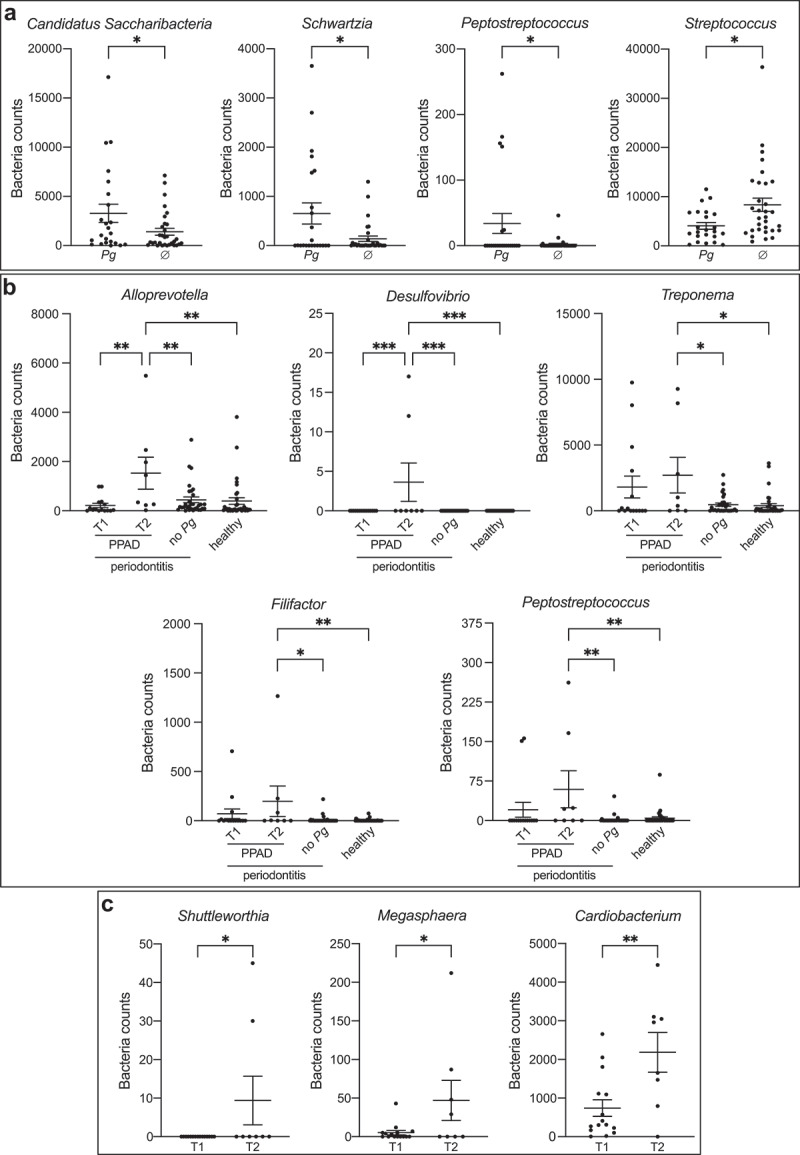
Selected bacteria genera influenced by (a) *P. gingivalis* positivity or (b and c) PPAD variant.

The presence of PPAD-T2 has led to an increased number of several bacterial genera: *Alloprevotella*, *Shuttleworthia*, *Megasphaera*, *Desulfovibrio, Cardiobacterium, Treponema, Filifactor,* and *Peptostreptococcus* (Figure 5). Interestingly, although the presence of PPAD-T2 enriched several groups, we have not seen any reverse impact where the increased citrullination proved to be detrimental and led to the loss of bacterial variety in the microbiome.

Salivary microbiota composition was characterized *via* 16S rRNA gene sequencing. Statistical significance between groups was evaluated with (a) unpaired t test, (b) One-way ANOVA with Tukey post-hoc and (c) unpaired t test. *p < .05, **p < .01, ***p < .001.

**Figure 7.**
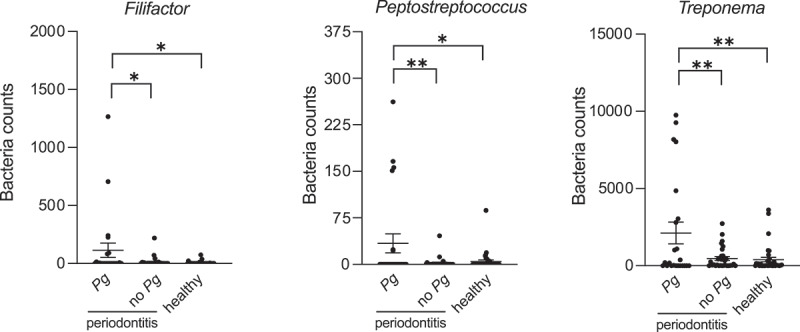
Selected bacteria genera influenced by *P. gingivalis* positivity. Salivary microbiota composition was characterized *via* 16S rRNA gene sequencing. Statistical significance between groups was evaluated with (a) unpaired t test, (b) one-way ANOVA with Tukey post-hoc and (c) unpaired t test. *p < .05, **p < .01, ***p < .001.

**Figure 6.**
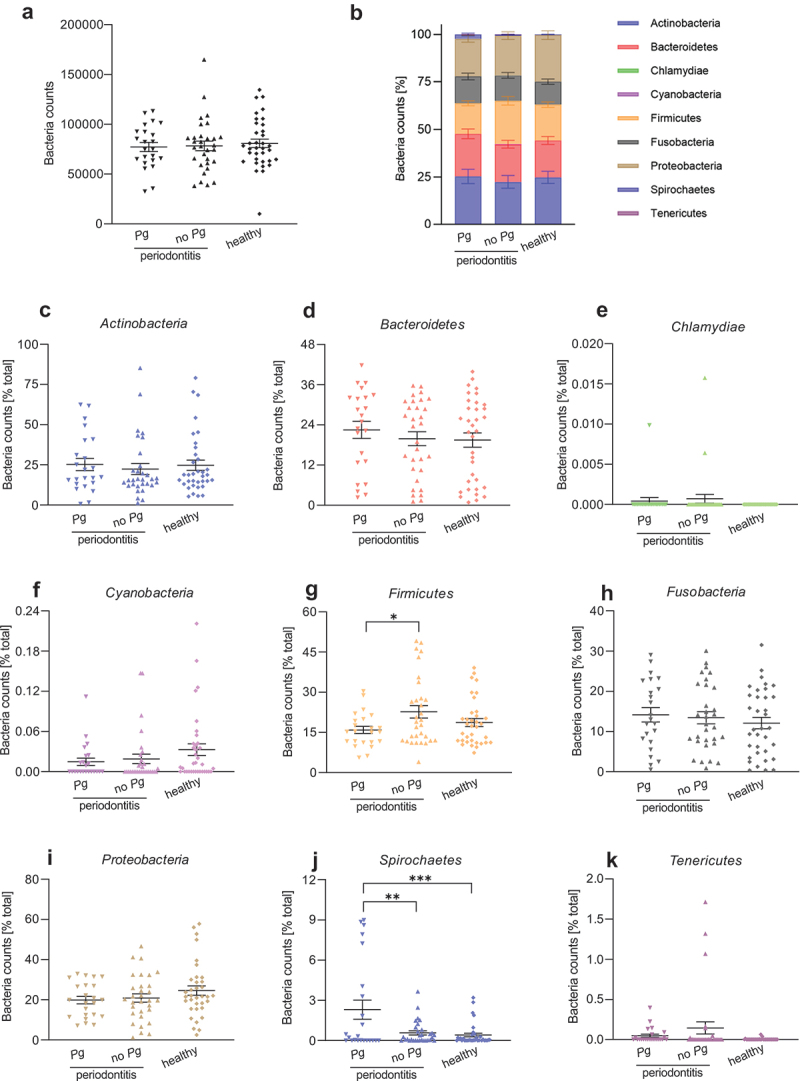
Influence of *P. gingivalis*-positivity on salivary microbiota: (a) bacteria counts, (b) biofilm composition as % of total bacteria counts, and abundance of (c) *Actinobacteria*, (d) *Bacteroidetes*, (e) *Chlamydiae*, (f) *Cyanobacteria*, (g) *Firmicutes*, (h) *Fusobacteria*, (i) *Proteobacteria*, (j) *Spirochaetes*, (k) *Tenericutes*. Salivary microbiota composition was characterized *via* 16S rRNA gene sequencing. Statistical significance between groups was evaluated with one-way ANOVA, with Tukey’s post-hoc analysis. *p < .05, **p < .01, ***p < .001.

Lastly, we have found that *Treponema, Filifactor, and Peptostreptococcus* genera were enriched in *P. gingivalis*-positive patients group (Figure 7). *P. gingivalis*-positive group was characterized by higher counts of *Spirochaetes* than both *P. gingivalis*-negative CP patients and healthy individuals, and lower counts of *Firmicutes* than patients bearing no *P. gingivalis* (Figure 6g,j).

## Discussion

Citrullination occurs in both physiological and pathological conditions serving a range of different functions. The five human peptidyl arginine deiminases exert distinct roles due to varying substrate specificity and their presence in different cellular compartments and tissues. Among them, PAD4 has the most extensive list of known substrates, including proteins from nuclear, cytosolic, cell surface, and extracellular domains. PAD4 impacts gene-expression [33,34], immune response [35], and can auto-citrullinate, thereby regulating the generation of the citrullinated proteins [36]. Apart from mammals, only a few species of bacteria belonging to the genus *Porphyromonas* express PAD enzymes capable of citrullination of proteins and peptides [37]. The best characterized is *P. gingivalis’* cysteine‐dependent PPAD. Although PPAD shares approximately 30% similarity with human PADs, it differs in specificity by efficiently citrullinating C-terminal arginine residues of target proteins, unlike mammalian enzymes that deiminate internal arginines [38]. *P. gingivalis* is prevalent in the subgingival plaque of CP patients and is mostly absent in healthy controls, identifying it as one of the primary pathogens implicated in the etiopathology of CP [39]. We recently discovered a PPAD variant (PPAD‐T2) harbouring three amino‐acid substitutions preceding the catalytic H236 residue (G231N/E232T/N235D) [40]. This variant demonstrates two-fold higher citrullinating activity, with weaker substrate binding affinity and higher turnover rates when compared to PPAD-T1. We have speculated that the presence of the T2 variant might increase the citrullination burden, potentially altering the microbiota composition and exacerbating the severity of CP.

Herein, we evaluated the prevalence of the PPAD-T2 variant in saliva samples from 56 patients with CP and 36 healthy volunteers. We also assessed the potential impact of increased citrullination capacity of *P. gingivalis* on the composition and robustness of salivary microbiota and the severity of periodontitis. Consistent with previous findings indicating that 30–90% of periodontitis patients harbor *P. gingivalis* [41–45], we found this keystone periodontopathogen present in approximately 45% of the CP patients in our study. This broad range in the prevalence of *P. gingivalis* across various studies may reflect differences in the populations examined, including factors such as age, geographic location, and oral hygiene practices. In our cohort, only 3% of healthy controls were *P. gingivalis*-positive, which is somewhat lower than other reports that have indicated a prevalence of at least 9% in healthy individuals [44,45]. It is also noteworthy that the percentage of periodontitis patients harboring *P. gingivalis* tends to increase with age, as pointed out by Manohar S [44]. The variation in detection rates between studies can also be attributed to the differences in sampling and detection methodologies. Real-time PCR is known for its high specificity and sensitivity, which makes it more reliable for detecting *P. gingivalis* than the traditional culturing methods. However, the real-time PCR performed on saliva samples might yield lower detection rates compared to subgingival DNA samples or culturing due to potential issues with DNA purity and retention in saliva [46]. Considering these factors, our finding that 45% of CP patients were positive for *P. gingivalis* is in line with the lower end of the range reported in the literature. This reinforces the importance of considering methodological and population-based differences when interpreting the prevalence of this pathogen across different studies.

Using real-time PCR, we confirmed that PPAD-T1 is predominant and present in around 68% of the *P. gingivalis*-positive patients, while the PPAD-T2 variant was detected in approximately 32% of cases. Interestingly, we have not seen any patient positive for both variants of PPAD. The fact that a previous study found 34% of patients carrying multiple *P. gingivalis* strains [47] raises a question if the PPAD variant is a determinant of strain colonisation.

Among the PPAD-T2-positive patients, the majority were females (6 patients to 2 male patients). The major limitation of the present study is the number of analysed patients. Therefore, population-based studies are needed in the context of autoimmune diseases for which females are at more risk. This includes RA, where female to male ratio is 3:1 [48]. Presence of the T2 PPAD variant could contribute to that increased risk, triggering the development of ACPAs [49].

16S rRNA gene sequencing revealed that several genera inhabiting subgingival space were differentially enriched among the study groups. Among the PPAD-T2 group we saw an increase in *Cardiobacterium*, a member of the HACEK group (*Haemophilus* species, *Aggregatibacter* species, *Cardiobacterium hominis, Eikenella corrodens* and *Kingella* species) implicated in the causation of endocarditis. *Eikenella* counts were also higher in PPAD-T1 and PPAD-T2 as compared to *P.gingivalis*-negative patients. Although infective endocarditis caused by the HACEK group is rather rare and accounts for 1.5% of cases [50], it is a severe disease with high mortality and a strong association with periodontitis [51]. Notably, we found that the relative abundance of bacteria *Desulfovibrio* genus was significantly elevated in patients possessing PPAD-T2 *P. gingivalis*. This gram-negative bacterium was also found to be more abundant in patients with RA [52] and had been suggested to have a strong association with Parkinson’s disease [53]. Additionally, the end-product of its metabolism (H_2_S) was shown to be toxic to oral epithelial cells [54] and might prime oxidative burst or attenuate apoptosis in polymorphonuclear leukocytes (PMNs) [55,56]. The increased abundance of the *Desulfovibrio* may be attributed to the increased citrullination in which deimination of the guanidine group of the arginine side chain results in the formation of citrulline and release of ammonia as a side product. As the average pH of the healthy gingival crevicular fluid is slightly acidic (pH = 6.64) [57] and during periodontitis drops to around 5.4 [58], the increased citrullination accompanied by ammonia production may increase the pH to levels more suitable for the *Desulfovibrio* growth.

Clinical parameters of periodontitis (BOP, CAL, and PD) were not affected by the PPAD variant. This suggests that the T1 variant is sufficient to maintain the virulence of *P. gingivalis* and that the higher activity of the T2 variant does not significantly influence the clinical progression of CP. In contrast, a recent study by Bereta et al. found that T2-PPAD correlated with a more severe presentation of CP [40]. This discrepancy may be due to the limited patient numbers in both studies, resulting in a low number of T2-positive patients available for analysis.

Interestingly, we identified several genera differentiating saliva microbiomes inhabited by PPAD-T1- and PPAD-T2-expressing *P. gingivalis* including *Alloprevotella*, *Corynebacteriaceae*, *Shuttleworthia*, *Megasphaera*, *Desulfovibrio, Cardiobacterium, Treponema, Filifactor, Peptococcus,* and *Synergistaceae*. Nevertheless, also this difference seemed to have no bearing on the severity of CP. Collectively, our findings revealed significant differences in the salivary microbiota among the studied groups, underscoring the need for further investigation into the potential impact of citrullination in oral microbiota and the development of chronic inflammatory diseases.

## Conclusions

Although the PPAD variant did not affect the clinical parameters of CP, it had a direct bearing on the salivary microbiota among the studied groups, underscoring the need for further investigation into the potential impact of citrullination on oral microbiota and the development of chronic inflammatory diseases. Future research should aim to include larger, more diverse populations to validate these findings and explore the mechanisms underlying the observed microbial differences. Additionally, longitudinal studies could provide insight into how these microbial communities evolve over time and contribute to disease progression.

In conclusion, while our study contributes to the understanding of the role of PPAD variants in periodontitis, it also raises important questions about the interplay between microbial composition and disease severity. Addressing these questions could lead to more effective strategies for managing periodontitis and its associated systemic conditions.

## Supplementary Material

Supplementary Material Figures legends.docx
