# Supplementary material for: Impact of increased Porphyromonas gingivalis peptidylarginine deiminase (PPAD) T2 variant allele on oral microbiota composition and severity of chronic periodontitis
Source: J Oral Microbiol. 2025 Mar 20;17(1):2479903. doi: 10.1080/20002297.2025.2479903 (PMC11926895; doi:10.1080/20002297.2025.2479903)
Supplement: Supplementary Material Figures legends.docx [file ZJOM_A_2479903_SM9152.docx]

SUPPLEMENT FIGURE 1

**Differences in microbiome composition assessed with beta-diversity**. Changes are assessed by the ordinate R function and visualized using Principal Coordinate Analysis (PCoA) plot based on Bray-Curtis distances between the samples of healthy and periodontitis groups (A) as well as between PPAD-T1 and PPAD-T2 among *P. gingivalis*-positive patients (B). Each dot represents one sample.

SUPPLEMENT FIGURE 2

**Selected bacteria genera (phylum Actinobacteria) abundance according to *P. gingivalis* positivity or PPAD variant.** One-way ANOVA with Tukey post-hoc test was used to assess the statistical significance, with *p<.05, **p<.01, ***p<.001.

SUPPLEMENT FIGURE 3

**Selected bacteria genera (phylum Bacteroidetes) abundance according to *P. gingivalis* positivity or PPAD variant.** One-way ANOVA with Tukey post-hoc test was used to assess the statistical significance with, *p<.05, **p<.01, ***p<.001.

SUPPLEMENT FIGURE 4 A

**Selected bacteria genera (phylum Firmicutes) abundance according to *P. gingivalis* positivity or PPAD variant.** One-way ANOVA with Tukey post-hoc test was used to assess the statistical significance with, *p<.05, **p<.01, ***p<.001.

SUPPLEMENT FIGURE 4 B

**Selected bacteria genera (phylum Firmicutes ─ continued) abundance according to *P. gingivalis* positivity or PPAD variant.** One-way ANOVA with Tukey post-hoc test was used to assess the statistical significance with, *p<.05, **p<.01, ***p<.001.

SUPPLEMENT FIGURE 5

**Selected bacteria genera (phylum Fusobacteria) abundance according to *P. gingivalis* positivity or PPAD variant.** One-way ANOVA with Tukey post-hoc test was used to assess the statistical significance with, *p<.05, **p<.01, ***p<.001.

SUPPLEMENT FIGURE 6 A

**Selected bacteria genera (phylum Proteobacteria) abundance according to *P. gingivalis* positivity or PPAD variant.** One-way ANOVA with Tukey post-hoc test was used to assess the statistical significance with, *p<.05, **p<.01, ***p<.001.

SUPPLEMENT FIGURE 6 B

**Selected bacteria genera (phylum Proteobacteria ─ continued) abundance according to *P. gingivalis* positivity or PPAD variant.** One-way ANOVA with Tukey post-hoc test was used to assess the statistical significance with, *p<.05, **p<.01, ***p<.001.

SUPPLEMENT FIGURE 7

**Selected bacteria genera (other) abundance according to *P. gingivalis* positivity or PPAD variant.** One-way ANOVA with Tukey post-hoc test was used to assess the statistical significance with, *p<.05, **p<.01, ***p<.001.
